# MVA-based vaccines are protective against lethal eastern equine encephalitis virus aerosol challenge in cynomolgus macaques

**DOI:** 10.1038/s41541-024-00842-y

**Published:** 2024-02-27

**Authors:** Brandon J. Beddingfield, Kenneth S. Plante, Jessica A. Plante, Scott C. Weaver, Sarah Bose, Clara Krzykwa, Nicole Chirichella, Rachel K. Redmann, Stephanie Z. Seiler, Jason Dufour, Robert V. Blair, Kathrin Endt, Ariane Volkmann, Nicholas J. Maness, Chad J. Roy

**Affiliations:** 1 Division of Microbiology, Tulane National Primate Research Center, Covington, LA USA; 2 Department of Microbiology and Immunology, University of Texas Medical Branch, Galveston, TX USA; 3 World Reference Center for Emerging Viruses and Arboviruses, Institute for Human Infections and Immunity, University of Texas Medical Branch, Galveston, TX USA; 4 Division of Veterinary Medicine, Tulane National Primate Research Center, Covington, LA USA; 5 Division of Comparative Pathology, Tulane National Primate Research Center, Covington, LA USA; 6 Bavarian Nordic GmbH, Fraunhofer Strasse 13, 82152 Martinsried, Germany; 7 Department of Microbiology and Immunology, Tulane School of Medicine, New Orleans, LA USA

**Keywords:** Viral infection, Experimental models of disease

## Abstract

MVA-based monovalent eastern equine encephalitis virus (MVA-BN-EEEV) and multivalent western, eastern, and Venezuelan equine encephalitis virus (MVA-BN-WEV) vaccines were evaluated in the cynomolgus macaque aerosol model of EEEV infection. Macaques vaccinated with two doses of 5 × 10^8^ infectious units of the MVA-BN-EEEV or MVA-BN-WEV vaccine by the intramuscular route rapidly developed robust levels of neutralizing antibodies to EEEV that persisted at high levels until challenge at day 84 via small particle aerosol delivery with a target inhaled dose of 10^7^ PFU of EEEV FL93-939. Robust protection was observed, with 7/8 animals receiving MVA-BN-EEEV and 100% (8/8) animals receiving MVA-BN-WEV surviving while only 2/8 mock vaccinated controls survived lethal challenge. Complete protection from viremia was afforded by both vaccines, with near complete protection from vRNA loads in tissues and any pathologic evidence of central nervous system damage. Overall, the results indicate both vaccines are effective in eliciting an immune response that is consistent with protection from aerosolized EEEV-induced disease.

## Introduction

Alphaviruses comprise a diverse genus of arthropod-transmitted viruses, including the encephalitic Venezuelan (VEEV), eastern (EEEV), and western equine encephalitis viruses (WEEV) that result in the pathology of the central nervous system in human and equine species^1^. Human infection results in fever, headache, malaise, and can progress to severe encephalitis. Case-fatality rates are estimated at 30–70% for EEEV, though this encephalitis can result in severe sequelae in survivors^2–4^. Due to the ease of which EEEV can be aerosolized^5^, as well as its capacity for incapacitating infected individuals, it is classified as a potential bioweapon, thus, the United States Center for Disease Control and Prevention classifies the North American variants of EEEV under the Select Agent program^6^. Natural infections do occur, along with other arboviruses such as dengue, West Nile, chikungunya and Zika among the emerging and reemerging infections that are predicted to continue appearing alongside ongoing climate change, including an outbreak of EEEV in the United States as recently as 2019^7,8^.

No current United States FDA-approved vaccine exists for human use against EEEV, though there is one for equine use as well as one investigational vaccine primarily used for laboratory personnel that shows moderate immunogenicity^9^. Live-attenuated and formalin-inactivated vaccines utilized for alphaviruses have limitations due to side effects or lack of efficacy. In the VEEV live-attenuated vaccine, TC-83, febrile illness symptoms are exhibited by a large portion of vaccinated individuals, which precludes use despite the production of neutralizing antibodies. Formalin-inactivated alphavirus vaccines are both poorly immunogenic and do not offer robust protection against aerosol-borne viral challenge^10–15^.

Modified Vaccinia Ankara (MVA) is a highly attenuated Vaccinia virus that is adapted to chicken embryo fibroblasts^16,17^. The MVA-BN platform is a further attenuated MVA, leaving it replication-deficient in humans and other mammals, including immunocompromised mice and non-human primates (NHPs)^18,19^, and is approved as smallpox and monkeypox vaccine in the US (JYNNEOS®), Canada (IMVAMUNE®) and the European Union (IMVANEX®). MVA-BN has been shown to be safe in humans^20–22^, and generates robust and durable immune responses^23,24^. The ability to simultaneously host several foreign gene components makes it an optimal candidate for a vaccine platform. This has been demonstrated for MVA-BN harboring filovirus antigens^25^, and has also been applied to encephalitic alphaviruses EEEV, WEEV and VEEV in murine models, resulting in robust protection from viral challenge. Monovalent and multivalent formulations of MVA-BN harboring antigens of all three aforementioned alphaviruses protect against homologous and heterologous intranasal and aerosol viral challenges in mice^26,27^.

In the work described here, the cynomolgus macaque (*Macaca fascicularis*) NHP model of small particle aerosol EEEV challenge was utilized due to its anticipated route of bioweapon distribution during an intentional viral release. Immunogenicity (binding and neutralizing antibodies and cellular responses) was examined prior to challenge with EEEV strain FL93-939, with monovalent (MVA-BN-EEEV) and trivalent (MVA-BN-WEV) formulations being evaluated for protective efficacy.

## Results

### Vaccine immunogenicity

**Fig. 1:**
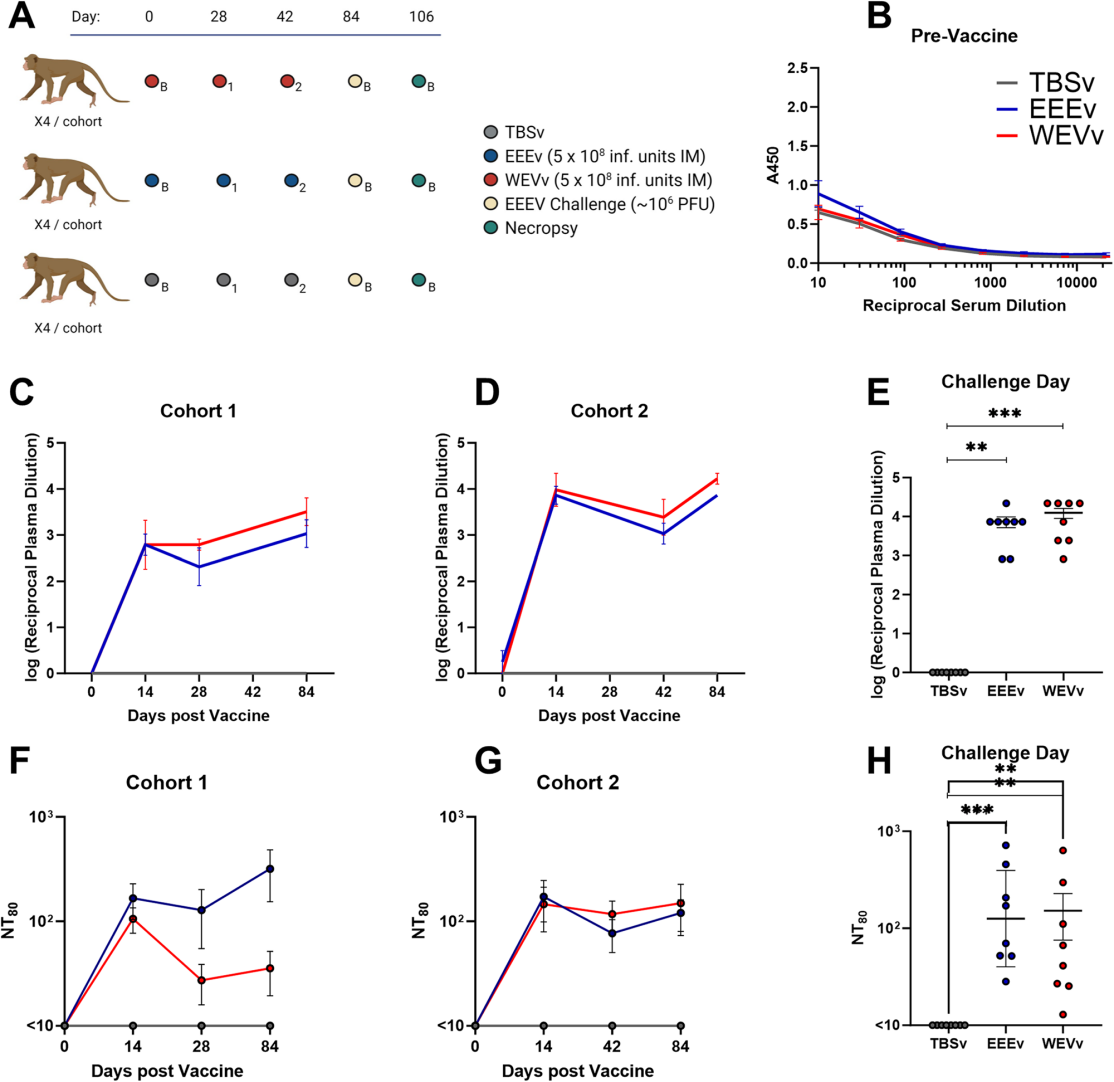
Assessment of antibody responses to vaccination. Cynomolgus macaques were vaccinated with TBS (TBSv), MVA-BN-EEEV (EEEv) or MVA-BN-WEV (WEVv) and subsequently challenged with EEEV via small particle aerosol inhalation at indicated time points (**A**). X4 = 4 animals, 1 = Cohort 1, 2 = Cohort 2, **B** = Both cohorts. Antibody responses were assessed via ELISA prior to vaccination (**B**) and post-vaccination using either a 28-day (cohort 1) or 42-day prime-boost schedule (cohort 2) (**C** and **D**, respectively). Group means +/− standard errors are shown. Individual final titers on the day of challenge (**E**). Neutralizing titer assessments by PRNT 80 were performed for both cohorts, with group means +/− standard errors shown (**F** and **G**), and individual final titers on the day of challenge (**H**). Group comparisons (area under the curve) were performed via Kruskal-Wallis test, with Dunn’s multiple comparisons test. Asterisks represent significant differences. **p < 0.01; ***p < 0.001. Study plan generated in Biorender.

Animals were vaccinated at day 0, with a boost at either day 28 (cohort 1) or day 42 (cohort 2), followed by EEEV challenge via small particle aerosol inhalation on day 84 (Fig. 1A). Before vaccination, animals were negative for EEEV-specific antibodies (Fig. 1B). Following an initial vaccination, both vaccine groups displayed a rapid, 3- to 4-log increase in anti-EEEV IgG titers that were significantly higher compared to the TBSv control group in both cohorts by day 14 (cohort 1: EEEv *p* = 0.0024, WEVv *p* = 0.0269; cohort 2: EEEv *p* = 0.0006, WEVv *p* = 0.0032), with an increase post-boost of a one half-to-full log (Fig. 1C, D). Titers for both cohorts were still significantly higher than in TBSv control animals at the day of challenge. Although the mean titer was slightly higher for WEVv than EEEv, the differences did not reach statistical significance, with a p-value > 0.999 (Fig. 1E).

Despite similar inter-cohort increases in binding IgG, cohorts displayed differing neutralizing immunogenicity kinetics as determined via PRNT, with cohort 1 showing more rapid drop-off for the WEVv group than the EEEv group after the first vaccination (Fig. 1F). However, cohort 2 displayed similar kinetics between vaccine types, with less drop-off than cohort 1 after the first vaccination. Cohort 2 also developed a lower day 84 titer for EEEv-vaccinated animals than WEVv-vaccinated animals; a reversal of that found in cohort 1 (Fig. 1G). Overall, group titers did not show significant differences between the NT_80_ values of WEVv and EEEv on the day of the challenge, but each vaccine induced a significantly higher titer than the mock vaccine (TBSv vs. EEEv *p* = 0.0003, TBSv vs. WEVv *p* = 0.0066, EEEv vs. WEVv *p* > 0.999) (Fig. 1H).

In contrast to humoral responses, T-cell responses, as assessed by ELISpot, showed no significant differences in the number of IFN-gamma-producing cells post-vaccination for both E1 and E2 proteins of EEEV between the vaccine and control groups (Supplementary Fig. 1).

### Post-challenge indications of protection

**Fig. 2:**
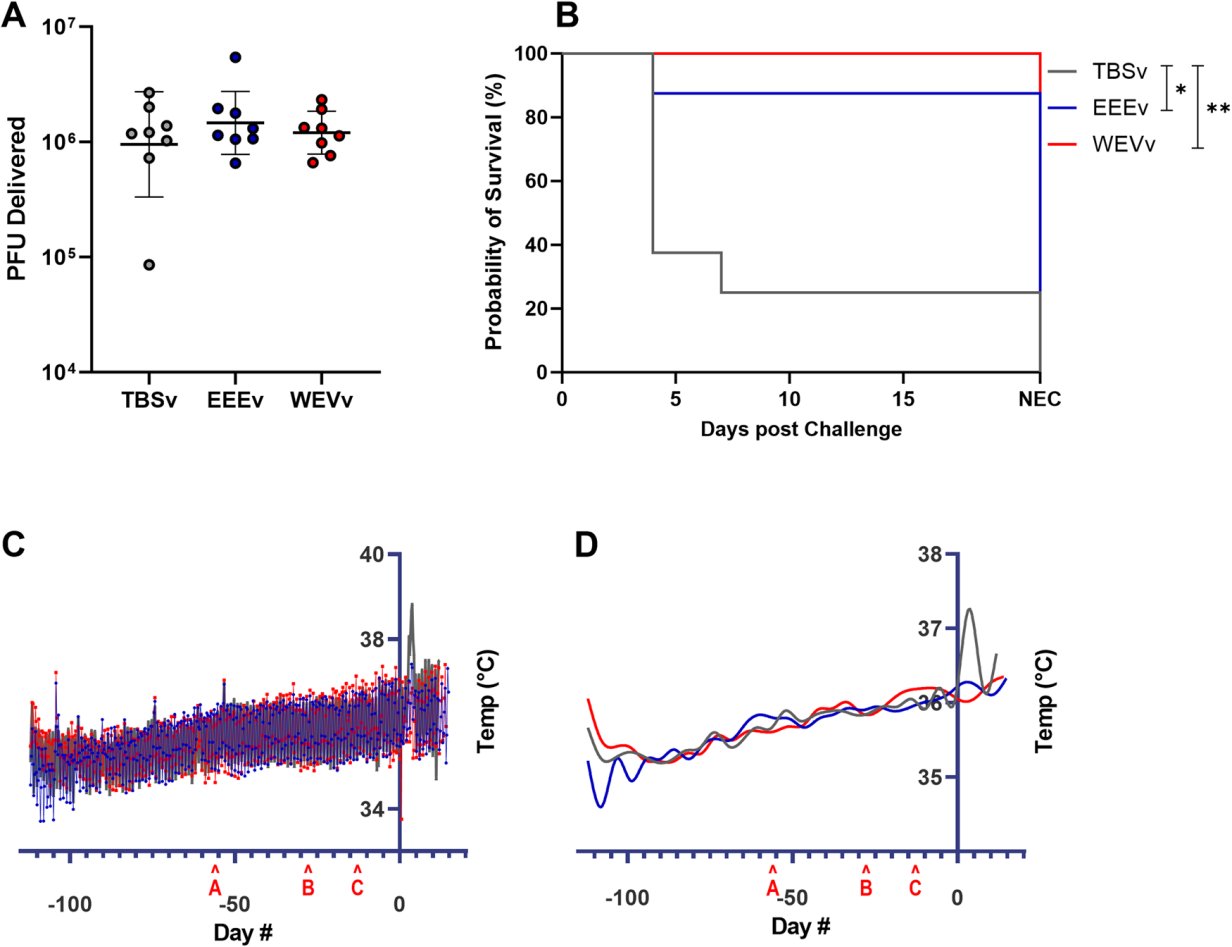
Challenge dose and post-challenge responses. The challenge was performed via a small particle aerosol of EEEV. Challenge dose delivered per animal determined via plaque assay (**A**) displayed as mean +/− SE. Kaplan-Meier plot of survival (**B**). NEC = day of necropsy. Group comparisons were performed using Mantel-Cox test. Asterisks represent significant differences. *p < 0.05; **p < 0.01. Temperature changes over time for each group as determined by implanted data loggers (**C**) with smoothed lines for easier visualization (**D**). Note the carat ‘A’ on X-axis represents the timing of the prime vaccination for both cohorts while ‘**B**’ and ‘**C**’ represent boost vaccinations for cohort 1 and 2, respectively. Time ‘0’ on X-axis and intersection with Y-axis represent the time of challenge.

Doses of aerosol virus inoculum delivered to each animal, calculated based upon the concentration of virus in the head-only chamber during each separate animal exposure as well as plethysmography data taken on each animal directly before the challenge, were determined to be within one log of the target dose of 10^7^ PFU/animal for all but one animal. The one exception was a mock-vaccinated control animal that received one log below the intended target dosage (Fig. 2A).

Survival to the end of the study was observed in 7/8 macaques that received EEEv and 8/8 macaques that received WEVv, compared with 2/8 macaques that received the mock vaccine (Fig. 2B). The macaque receiving the EEEv vaccine that did not survive was boosted day 28 post prime vaccination. The TBSv group animals displayed the vast majority of clinical signs expected post-challenge, with 6/8 displaying a combination of tremors or other neurological signs, increased respiratory effort and decreased activity. These indications resulted in recommendations of euthanasia by the veterinarians, typically 4 days post-challenge. The EEEv group demonstrated much less severe signs of disease, with only 1/8 animals reaching euthanasia criteria after displaying severely increased respiratory effort and decreased activity. None of the WEVv animals required euthanasia, with only 1/8 animals displaying a mildly increased respiratory rate. No EEEv or WEVv animals displayed any neurological indications of disease.

All animals were implanted with data loggers, with data continuously collected during the in-life portion of the study. Differences in core temperature were observed between vaccinated and mock vaccinated groups, with TBSv animals presenting increased temperatures two days post-challenge (Fig. 2C, D). Temperature increases were approximately 1–1.5 °C in the TBSv group, with no change seen in the protected animals of the EEEv and WEVv groups.

### Viral load post challenge

**Fig. 3:**
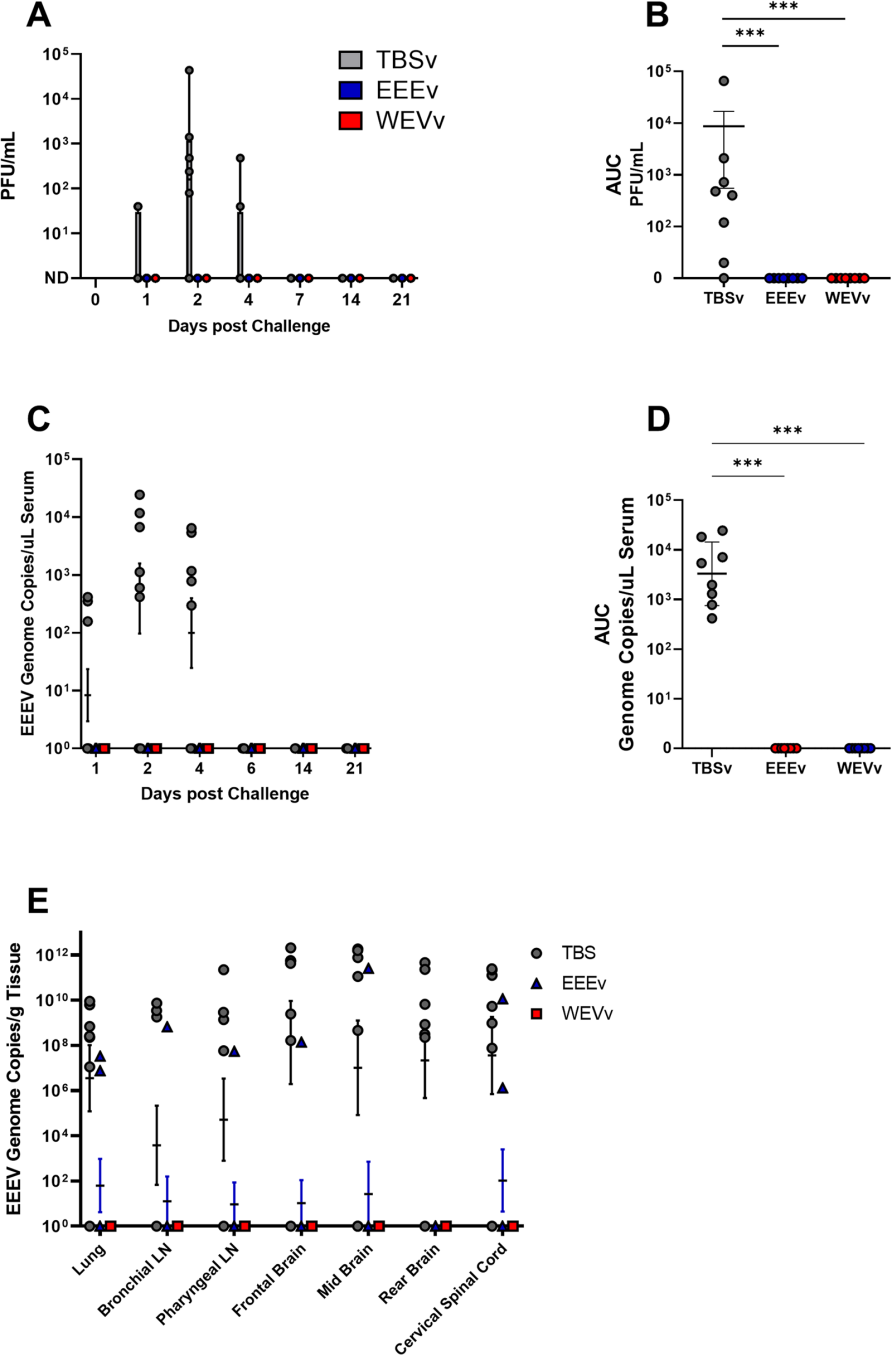
Viral loads post small particle EEEV challenge of cynomolgus macaques. Plaque assays and qPCR were used to assess virus post-challenge. Group data (**A**, **C**) is displayed along with AUC analysis (**B**, **D**) for live virus (**A**, **B**) or viral genome content (**C**, **D**) in serum. Virus in tissues as determined by qPCR (**E**). Group comparisons were made using Kruskal-Wallis test. Asterisks represent significant differences from TBS vaccine group. ***p < 0.001. Data is displayed as mean +/− SE.

Though survival is our primary endpoint for the assessment of vaccine efficacy, the presence of viremia post-challenge is an important secondary concern. To assess this, we utilized plaque assays and RT-qPCR. All TBSv animals displayed viremia at some point before euthanasia, while no EEEv or WEVv animals had any detectable infectious virus present (Fig. 3A). TBSv animals displayed variable patterns of viremia, though most had a peak at 2 days post-challenge, with two animals viremic on day 4 and no viremic animals on day 7. Comparisons of area under the curve (AUC) for viremia were highly significant (*p* < 0.001) for both vaccine groups compared to mock vaccinated animals (Fig. 3B).

Viral RNA (vRNA) content in both blood and tissues (frontal, mid and rear brain, lung, cervical spinal cord, mediastinal lymph node, and bronchial lymph node) was assessed via RT-qPCR. vRNA was present in serum samples from all control animals, while none of the vaccinated animals showed evidence of vRNA by this method, displaying a similar pattern to infectious virus described in the previous paragraph (Fig. 3C). Differences between the controls and both vaccine groups were highly significant (*p* < 0.001), while no differences were observed between vaccine groups (Fig. 3D). For the control animals, tissues showed a pattern similar to blood, with high levels of vRNA detected in all tissues, ranging from 10^8^ to 10^12^ genome copies/g tissue in many animals. vRNA was also detected in the tissues of some animals in the EEEv group, though detection was uncommon. Two EEEv animals had approximately 10^7^ copies/g in the lung, and two had moderate to high levels of vRNA in the cervical spinal cord. One EEEv animal, which succumbed to disease, had vRNA in all tissues examined. No animals in the WEVv group displayed any evidence of vRNA in tissues (Fig. 3E).

### Histopathology

No significant gross abnormalities were noted in any of the twenty-four animals examined. The central nervous system (brain and spinal cord) was grossly normal in all animals examined. Eight of the animals (5/8 TBSv, 1/8 EEEv, 2/8 WEVv) had minimal gross pulmonary lesions consisting of small, localized regions of either tan or red discoloration. Three of the animals (1/3 EEEv, 2/3 WEVv) had multifocal, geographic, red areas in the spleen (interpreted as segmental congestion). Lymphoid hyperplasia of the mesenteric lymph nodes (MLN) and bronchial lymph nodes (BLN) varied from mild to moderate and was identified in six animals each (MLN: 1/6 TBSv, 3/6 EEEv, 2/6, WEVv and BLN: 2/6 TBSv, 3/6 EEEv, 1/6 WEVv).

**Fig. 4:**
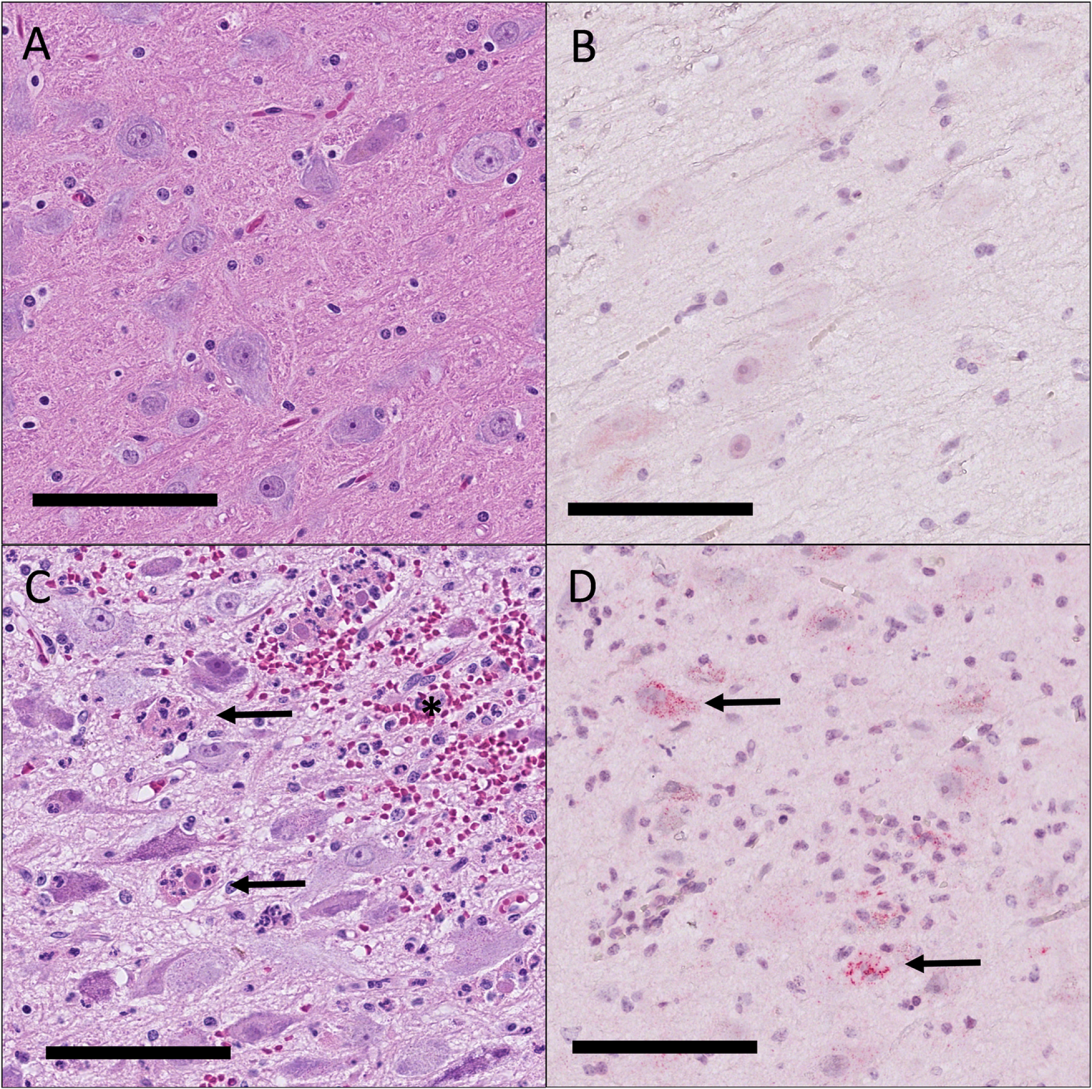
Histopathology and immunohistochemistry in EEEV-challenged NHPs. Brainstem, representative sample. Immunized animals (**A**, **B**) had minimal to no encephalitis following EEV challenge (**A**), and immunohistochemistry for EEEV (**B**) was often negative. In contrast, animals that received the placebo (**C**, **D**) had characteristic EEEV encephalitis with hemorrhage (asterisks), neuronal necrosis, and neuronophagia (**C**, arrows). Immunohistochemistry for EEEV (**D**) confirmed the presence of viral antigen (red) at sites of encephalitis (arrows). Bar = 100 μm.

Histopathologic changes included inflammation in the central nervous system (CNS) and lungs, and lymphoid hyperplasia within the bronchial lymph node and lungs (Supplementary Table 2). There were significant differences in the severity of CNS inflammation among animals within the study suggesting a strong effect based on the experimental group. Overall, EEEv and WEVv animals had much less histologically detectable disease by both H&E (Fig. 4A) and IHC (Fig. 4B) than those of the TBSv group (Fig. 4C, D). Four animals that did not survive the challenge (3 TBSv and 1 EEEv), exhibited moderate inflammation characterized by widespread CNS inflammation with characteristic lesions of EEEV encephalitis. Three animals, all TBSv, exhibited mild inflammation characterized by CNS inflammation in more than two tissue sections that also contained neutrophilic infiltration consistent with EEEV encephalitis. Six animals (1/6 TBSv, 2/6 EEEv and 3/6 WEVv) exhibited minimal inflammation characterized by one or two areas of CNS tissue with perivascular inflammation composed of mononuclear cells (and lacking neutrophilic infiltrate). The remaining 11 animals had no evidence of inflammation in any of the CNS regions examined (frontal cortex, parietal cortex, temporal cortex, occipital cortex, brainstem, cerebellum, and cervical spinal cord). Two animals, 1 TBSv and 1 WEVv (minimal and no CNS inflammation respectively), had focal cortical areas suggestive of a healed infarct. Given the chronicity, stage of repair (mostly complete), and lack of active inflammation at these sites it was not possible to determine if these lesions occurred before or after EEEV exposure.

Bronchial lymph node hyperplasia varied from absent to moderate without a clear association with pulmonary or CNS inflammation, making an experimental group effect unlikely for this microscopic change. No significant differences were noted in pulmonary pathology between animals. All animals in the study exhibited minimal to mild multifocal interstitial inflammation that affected <5% of the examined sections. Therefore, a group effect is considered unlikely and the role of EEEV exposure is difficult to determine because this level of inflammation is commonly seen in NHP regardless of experimental status.

Immunohistochemistry for EEEV was performed on three brain regions for each of the 24 animals. The parietal cortex, temporal cortex, and cerebellum with brainstem were selected for evaluation due to having the highest-level inflammation across the study set. These three regions were evaluated in 23/24 animals, and in the remaining animal, the frontal cortex was evaluated instead of the parietal cortex due to the higher level of inflammation in this region in this animal. EEEV antigens were detected by IHC in all three experimental groups; however, antigen detection only correlated with pathology in the TBSv group (Fig. 4B, D).

### Correlates of protection

**Fig. 5:**
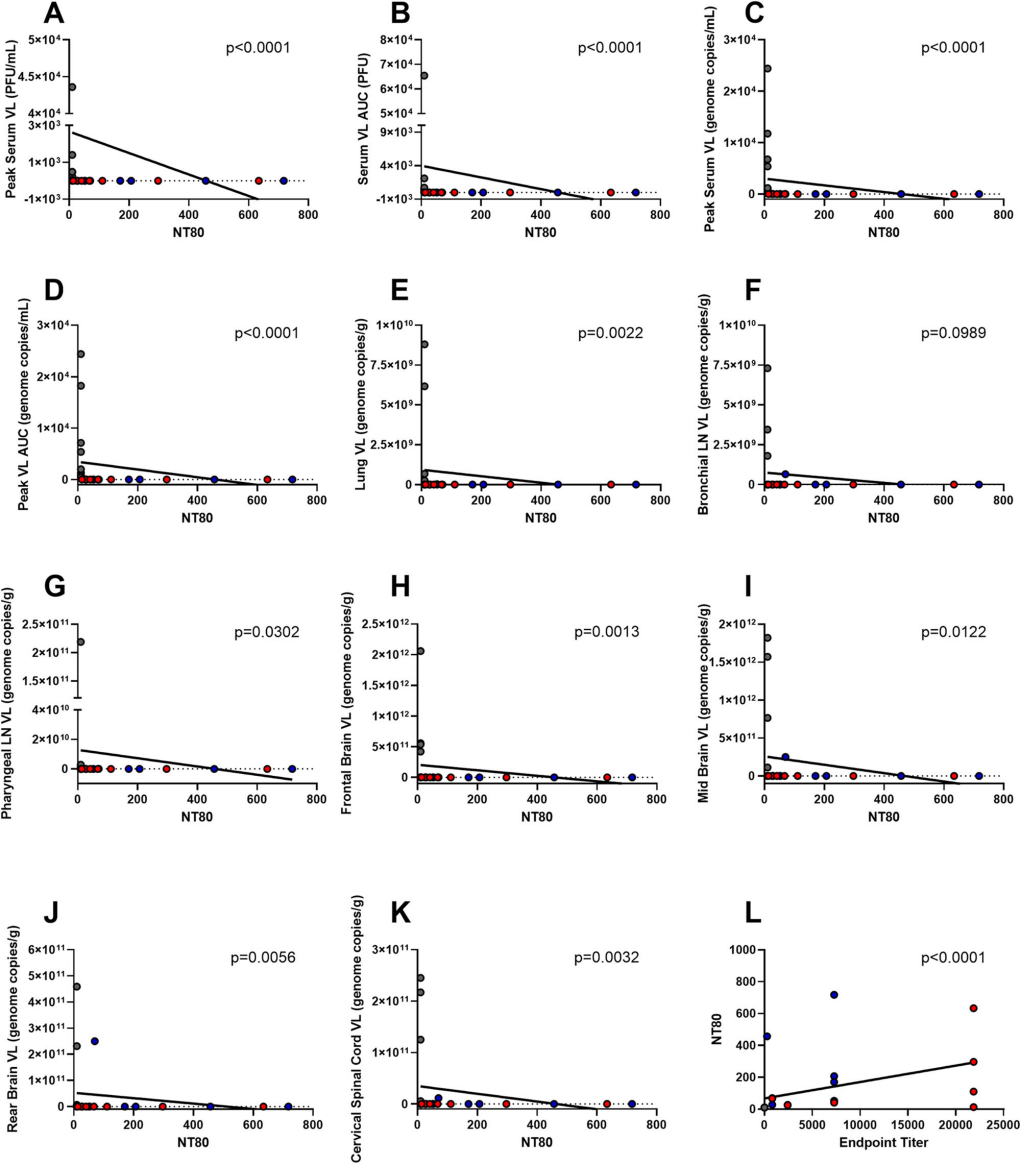
Antibody Correlations. NT_80_ and viral loads of serum (**A**–**D**) and tissues (**E**–**K**). Binding and neutralizing antibody correlation (**L**). Analysis was performed via the nonparametric Spearman correlation, with *p*-values listed above each figure for that comparison.

In order to determine if protection was due to post vaccination antibody levels, we analyzed correlation of neutralizing antibody levels, as measured by NT_80_, with viral loading of serum and tissues. NT_80_ correlated with peak viral loads by plaque and qPCR in serum, as well as AUC of each metric, with a *p* value of <0.0001 for each of these (Fig. 5A–D). Neutralizing antibody levels also correlated with viral loads as assessed by qPCR in tissue at necropsy, with p values ranging from 0.0013 to 0.0302. The sole exception to the correlates was that of the bronchial lymph node, with a *p* value of 0.0989 (Fig. 5E–K). NT_80_ also correlated with endpoint titers as assessed by ELISA, with a *p* value of <0.0001 (Fig. 5L).

### Hematology and clinical chemistries

Hematology and clinical chemistry parameters were examined during the challenge phase of the study. Neutrophil, basophil, monocyte, and overall WBC counts were elevated by day 4 post-challenge in the TBSv group, in keeping with prior work seeing increases in granulocyte count in lethally infected macaques^28^. This elevation was not seen in vaccinated NHP, though these differences did not reach statistical significance. Hematocrit was significantly decreased in the control group post-challenge compared to the WEV group (post-challenge day 4, *p* = 0.0227), although since this was also significantly different at the time of challenge (*p* = 0.0227), this may be the result of natural animal variation, though it did continue to trend downward in control animals. Hemoglobin trended downward in control animals as well, without differences reaching statistical significance (Supplementary Fig. 2).

Clinical chemistries were also examined post-challenge. On day 7 post-challenge, potassium was reduced significantly in the control group compared to the WEV group (*p* = 0.0200), while bilirubin was significantly elevated in the control group compared to both vaccine groups (TBSv vs. EEEv *p* = 0.0223, TBSv vs. WEVv *p* = 0.0221). This could reflect the very small group numbers in the control group, since at this time post-challenge only 2 control animals had survived. CO_2_ concentrations trended down on day 7 as well in control animals, though this could be due to group size changes as well and did not reach significance. On day 4 post-challenge, C-reactive protein was significantly elevated in the TBSv group compared to the WEVv group (*p* = 0.0220). Blood Urea Nitrogen (BUN) and creatinine trended slightly higher in the control group than either vaccine group but did not reach significance (Supplementary Fig. 3).

## Discussion

A total of 24 adult cynomolgus macaques in three equally distributed groups (*n* = 8) were vaccinated or sham-vaccinated with either MVA-BN-EEEV (EEEv), MVA-BN-WEV (WEVv, or TBS (TBSv), respectively, in two cohorts. All animals were biosampled for measurement of EEEV-specific immune responses, both humoral and T cell-mediated, throughout the vaccination period. Thereafter, all animals were challenged with EEEV FL93-939 by small particle aerosol to determine efficacy of both vaccine formulations. This inhalation challenge route is consistent with an anticipated route of infection during an intentional bioweapon release of EEEV, as well as a possible source of a laboratory-acquired infection. Animals were monitored for physiologic response via implantable wireless telemetry throughout the study. All animals that succumbed to EEEV disease or survivors of the observational period were euthanized. Following necropsy, selected tissues were assessed via histopathology/IHC as well as nucleic acid analysis for viral loading in both tissues and blood.

Immunogenicity in response to vaccination was overall robust in the groups vaccinated with either MVA-BN-based vaccine. The ELISA analysis revealed a rapid increase in anti-EEEV IgG antibody titers after the first vaccination. When analyzed via area under the curve of the dilution series or endpoint titers, the multivalent vaccine showed a higher mean titer of antibody compared to the monovalent vaccine, but this did not reach significance. Both cohorts showed an increase in antibody levels shortly after vaccination, followed by an increase after boost that was maintained for at least 84 days post-prime, to levels similar to or slightly above those achieved 14 days post-prime. The cohort receiving the day 42 boost responded with a higher day 84 titer than the 28-day boost to the WEVv vaccine, indicating that this may be the preferred schedule. The differences in response may also be due to the heterogeneity of the macaque population under study. The PRNT analysis provided confirmatory congruence with the ELISA data, demonstrating an overall increase in neutralizing antibody titers following boost. There was a similarly high level of neutralizing antibody development 84 days post-initial vaccination between the two vaccine types. Titers were similar in animals between the EEEv and WEVv vaccine regimens, but both EEEv and WEVv groups demonstrated significantly higher neutralizing titers than the TBSv group. In this study, all NHP seroconverted after the first vaccination, while in previous murine studies (Henning et al.) with EEEv and WEVv, complete seroconversion in terms of neutralizing antibodies could not even been detected 14 days after secondary EEEv or WEVv administration. However, although some vaccinated mice did not exhibit a measurable neutralizing antibody response prior to challenge, all animals in the EEEv and WEVv groups survived the otherwise lethal EEEV V105-00210 aerosol challenge^27^, which agrees with our findings of significant protective efficacy.

In contrast to these prior murine studies, correlations were found between viral loading of macaques and high antibody levels. In addition, neutralizing antibody levels generally correlated well with binding antibody levels. This relationship is not perfect; however, as one individual vaccinated with the EEEv vaccine who succumbed to infection had a mid-range level of binding antibody but a low level of neutralizing antibody, while another who survived had a high level of neutralizing antibody but lower levels of binding antibodies. This is in line with heterologous responses expected within the macaque population. A caveat to the low viral load in survivors is the fact that surviving animals had tissue samples taken at a later time point than those that underwent euthanasia due to signs of disease, thus potentially allowing for viral levels to artificially lower during this time. A study incorporating earlier time points for euthanasia of vaccine recipients was not feasible for this study due to limitations inherent in the study of NHP but should be considered in future work.

The ELISpot analysis showed a less robust pattern of T-cell development. Though a minor increase in antiviral T-cells was seen in both vaccine groups, these responses never achieved statistical significance relative to the sham vaccine. High-level anti-CD3 control responses were shown for some time points, but the highly variable nature of the responses made interpretation of this data difficult and inconclusive. This pattern was observed for both E1 and E2 EEEV peptide pools. The high background present in the assay, as seen in the number of spots present in the TBSv samples, may preclude detection of a relevant T cell response, and should be studied further in the future, including flow cytometry-based analysis of immune activation. Nonetheless, these data potentially indicate neither vaccine elicits robust antiviral T-cell responses. This is surprising, as MVA-vectored vaccines have typically induced cellular immunity well in prior studies^29,30^. In this study, peptide pools were used that are similar to those used in prior work in mice with the same vaccines, where effective induction of T cell responses by the same WEVv was demonstrated^27^. Similar to the mouse data, T cell responses have also been noted in human participants of a Phase 1 clinical trial (NCT04131595, manuscript in preparation) using WEVv, lending credence to the idea that assay failure may be the reason for no measurable T cell responses above TBS background in this study. Alternatively, species differences may contribute, which remains to be seen and should be further examined in future work.

We analyzed post-challenge EEEV viremia by both plaque assay and RT-qPCR. All TBSv animals had infectious virus and vRNA detected in blood at one or more time points prior to euthanasia, while no vaccinated animal had either at any time point, indicating a robust anti-viral immune response. This is in keeping with prior work done in mice^27^. Samples analyzed post-euthanasia by RT-qPCR revealed the presence of vRNA in all tissues analyzed for almost all control animals. One to two animals in the EEEv-vaccinated group showed the presence of vRNA in tissues except for the rear brain, with the animal succumbing to infection having detectable vRNA in multiple tissues. No animals that received the WEVv vaccine had any vRNA in any tissue. Timing differences cannot be ruled out, as the animals that made it to the end of the study period were necropsied at a later time point post-challenge than those who did not survive the challenge.

Post-challenge, two of the eight animals in the control group survived to planned necropsy (25%), while the remaining six of the eight control group animals met euthanasia criteria approximately four days post challenge. One control animal that survived the challenge received a lower challenge dose than expected. Seven of the eight animals that received the EEEv vaccine survived (87%), and all animals receiving the WEVv vaccine survived (100%), indicating both vaccines significantly increased the chances of survival post-challenge. Both vaccines achieved the primary study endpoint of protection from death from EEEV FL93-939 infection. In prior work in mice, heterologous challenge by aerosol with EEEV V105-00210 showed protection as well, indicating a high likelihood of multi-strain protection with these vaccine formulations^27^. Upon necropsy, CNS pathology was apparent in sham-vaccinated animals, demonstrating characteristic encephalitis marked by neutrophilic inflammation and neuronophagia in most animals in this experimental group. There was minimal to no CNS pathology in all vaccinated animals, except for one animal that received the EEEv vaccine and succumbed to the disease.

In comparison with other work done in NHP in pursuit of an effective EEEV vaccine, this vaccine induces a humoral response on par with others including monovalent VLP-based vaccines against EEEV, as well as a trivalent preparation. NHP survival was slightly more robust for the VLP vaccines than the EEEv here, but the same level of protection against death was afforded by WEVv here^31^. Complete protection has also been shown in NHP using alphavirus replicon-based vaccines, with a similar level of humoral response noted. These vaccine types demonstrated protection that takes some time to form, with much less protection shown at 1.5 months post-prime than at 2 months in mice. The rapid production of neutralizing antibodies with the MVA-BN-based vaccines demonstrated in our study is a potential benefit over this replicon-based strategy^32^. Much higher humoral responses, on the order of two logs, are afforded by this vaccine than chimeric vaccines based upon the Sindbis virus, with both EEEv and WEVv formulations providing more complete protection^33^.

In summary, the vaccines (EEEVv, WEVv) we evaluated induced an immune response in cynomolgus macaques that was consistent with the robust protection required to prevent encephalitis and death consequent to aerosolized EEEV challenge. Both vaccines generated a rapid neutralizing antibody response, with most vaccinated animals surviving the challenge with minimal viremia, tissue viral load and virally induced pathological consequence. WEVv was the more robust of the two formulations based upon 100% survival. Together with its potential to protect against heterologous challenge as seen in mice (26), although not evaluated in this study, these results warrant further development of WEVv (MVA-BN-WEV), which is currently under investigation in a Phase 1 clinical trial (ClinicalTrials.gov Identifier# NCT04131595).

## Methods

### Experimental design

MVA-BN-EEEV and MVA-BN-WEV are based on MVA-BN® (ECACC cat no. V00083008)^18^. MVA-BN-EEEV was generated using an inserted, codon-optimized, sequence of E3-E2-6K-E1 envelope proteins into MVA-BN under the control of pox promoters. MVA-BN-WEV was generated similarly, using insertions for EEEV, WEEV, and VEEV simultaneously. The design and generation of vaccines were described previously^26^. This experiment was undertaken to determine the immunogenicity and protective efficacy of MVA-based vaccine formulations against EEEV aerosol challenge. Twenty-four (24) cynomolgus macaques were randomly assigned to three experimental groups. Group I animals (*n* = 8) received the MVA-BN-EEEV monovalent vaccine, Group II animals (*n* = 8) received the MVA-BN-WEV multivalent (EEE, VEE, WEE) vaccine, and Group III animals (*n* = 8) received the sham vaccine (tris buffered saline). Hereafter, MVA-BN-EEEV will be referred to as EEEv, MVA-BN-WEV will be referred to as WEVv, and sham vaccine will be referred to as TBSv. Animals of all groups were prime-vaccinated on day 0 and boosted at day +28 (cohort 1) or day +42 (cohort 2) via the intramuscular route using a 22 gauge, 1-inch needle on a 1 mL syringe. Schedule differences were due to weather conditions preempting work. On day +56, all groups were scheduled to be challenged via aerosol with EEEV, but this was extended to day +84 due to unforeseen weather conditions. For a minimum of 22 days post-experimental infection, animals were observed for clinical signs of illness and monitored for viremia and changes in hematology, serum chemistries, and temperature to determine the protective efficacy afforded by the experimental vaccine candidates. To accommodate ABSL3 housing limitations, the experiment was divided into two cohorts of 12 animals each with an equal number of representatives from each vaccine group with procedures following the same schedule. Timing of euthanasia within each 12-animal cohort was further staggered to limit necropsy to no more than 3–4 animals per day at 22 days post-challenge.

### Animals

Twenty-four age-matched, female, cynomolgus macaques (*Macaca fascicularis*) weighing 2.75–4.3 kg and free of simian immunodeficiency virus (SIV), simian type D retrovirus (SRV), simian T-Lymphotropic virus (STLV) and alphavirus antibodies were used. Split-sex cohorts of animals were ideally desired for assignment to this study; however, a NHP shortage precipitated by extrinsic, uncontrollable events precluded the procurement of animals with these desired characteristics.

The vivarium light cycle was set at 12:12 h of light: dark. All animals were fed a Purina LabDiet nonhuman primate diet, which is nutritionally complete. The Purina Mills diet was supplemented with a variety of fruits and vegetables at a minimum of three times each week. Water was provided *ad libitum*.

The study was approved by the Institutional Animal Care and Use Committee at Tulane University, and all animals were handled in accordance with guidance from the American Association for Accreditation of Laboratory Animal Care. Animals were monitored daily prior to vaccination, twice daily after vaccination and at least three times daily post-viral challenge. Biosamples were collected pre-exposure as well as post-exposure and at necropsy. Physical examinations were performed daily after exposure, and euthanasia occurred either on upon reaching humans assessment guidelines from study endpoint or at the termination of the post-challenge observation period. A full necropsy was performed on every animal involved in this study. Anesthesia used for animal procedures was administered via bolus intramuscular injection of ketamine. Euthanasia was performed via intravenous injection of sodium pentobarbital administered on anesthetized animals. The procedures used for anesthesia, experimental endpoints, and euthanasia of study animals followed tenets of the ARRIVE reporting guidelines^34^.

### Challenge virus

Challenge virus stocks were prepared at the World Reference Center for Emerging Viruses and Arboviruses (WRCEVA) at the University of Texas Medical Branch (UTMB) in Galveston, Texas, USA. Virus stock consisted of supernatant derived from infection of, and propagation with, monolayers of VeroE6 cells. The supernatant from cells post-visible cytopathic effect was removed and cleared via centrifugation. Challenge material was titrated by plaque assay on VeroE6 cells.

### Biotelemetry

Animals were subcutaneously implanted with Star-Oddi data loggers (DST milli-HRT ACT) in the dorsum during the Baseline Phase to allow for uninterrupted physiological monitoring. Implants recorded up to three parameters (temperature, heart rate, and activity) and were removed following euthanasia for data recovery and analysis.

### Vaccination Procedures

Animals were vaccinated intramuscularly with 0.5 mL of either TBS (*n* = 8), or 5 × 10^8^ infectious units of either monovalent MVA-BN-EEEV (*n* = 8) or multivalent vaccine MVA-BN-WEV (*n* = 8).

### Immunogenicity assessment

An enzyme-linked immunosorbent assay (ELISA) was used to assess antibody binding responses. Plates coated with whole inactivated EEEV antigen (Hennessey Research, KS, USA) at a 1:40 dilution in carbonate-bicarbonate buffer at pH 9.2 were incubated with serial 1:3 dilutions of plasma for one hour at room temperature (RT). Plates were washed 3X with PBS + 0.05% Tween-20 (PBS-T) and a secondary anti-monkey IgG-HRP (PA1-84631, Invitrogen, IL, USA) at a 1:25,000 dilution was added to each well and incubated at RT for one hour. Following incubation, plates were washed 3X with PBS-T and developed with 1-step Ultra TMB Substrate Solution (Thermo Fisher, MA, USA) for 15 min in the dark. Following development, the reaction was stopped, and plates were read at 450 nm and endpoint titers were calculated.

Assessment of neutralizing antibody responses was performed via Plaque Reduction Neutralization Testing (PRNT). The serum was heat-inactivated for 30 min at 56 °C prior to assays. Serum was serially diluted 1:2 in serum-free Dulbecco’s Modified Eagles Medium (DMEM) and incubated for one hour at 37 °C/5% CO_2_ with approximately 25 PFU of replication-competent EEEV. After incubation, 125uL of serum/virus mixtures were added to each well of 24-well plates containing monolayers of VeroE6 cells and incubated for one hour at 37 °C/5% CO_2_ with rocking every 15 min. Following adsorption, the inoculum was removed, and the monolayers were overlayed with MEM + 2% FBS + 1.2% Avicel. Plates were allowed to incubate at 37 °C/5% CO_2_ for two days. After development, plates were fixed with formalin and stained with 0.1% crystal violet. PRNT titers were calculated as the amount of serum in a solution necessary to neutralize 80% of the live virus (NT_80_).

Vaccine-induced T-cell responses were assessed via Enzyme-Linked Immunospot (ELISpot) assay. Separated PBMCs were stimulated overnight on pre-coated plates (cat# 3421M-4HPT, Mabtech, OH, USA) with a final concentration of 1 ug/mL per peptide in separate pools of peptides spanning the E1 or E2 protein per sample, a negative control of equivalent amounts of DMSO in media, or a positive control of anti-CD3 mAb at a 1:1,000 dilution. After overnight incubation, IFN-gamma-secreting cells were detected by following the manufacturer’s instructions, using the detection antibody at 1 ug/mL and the streptavidin-HRP at a 1:1,000 dilution. Data were calculated as the number of spots per million cells.

### Virus and cells

Virus used for animal inoculation and PRNT was strain EEEV FL93-939, generated from a cDNA clone described previously^35^ and prepared on subconfluent VeroE6 cells (ATCC# CRL-1586). VeroE6 cells were used for live virus titration of biological samples and were maintained in DMEM (#11965092, Thermo Scientific, USA) with 10% FBS.

### Aerosol challenge

On day 84 post-first vaccination, anesthetized macaques were challenged with virulent EEEV strain FL93-939 using a 16-liter head-only dynamic inhalation exposure system (Automated Bioaerosol Exposure System, AeroMP, Biaera Technologies, Hagerstown, MD)^36^. Prevailing concentrations of the virus in the head-only chamber were determined via sampling in an all-glass impinger. Coupled with data derived from plethysmography taken directly prior to the challenge, a dosage of virus delivered was determined for each animal.

### Quantification of live virus in blood

Viremia was measured post-challenge via plaque assay. Monolayers of VeroE6 cells were overlayed with serial 10-fold dilutions of serum in serum-free DMEM. Following incubation at 37 °C/5% CO_2_ for one hour with rocking every 15 min, the inoculum was removed, and monolayers were overlayed with MEM + 2% FBS + 1.2% Avicel. Plates were incubated at 37 °C/5% CO2 for two days. After development, plates were fixed with formalin and stained with 0.1% crystal violet.

### Quantification of viral RNA in blood and tissues

Viral genomic RNA from tissues and blood was extracted utilizing the Qiagen RNeasy mini kit (Qiagen, Germany) per the manufacturer’s protocol. For blood, 100 uL of serum was used for extraction, while 50 mg of tissue was used for each extraction. RNA was converted to cDNA using the iScript cDNA Synthesis Kit (Biorad, CA, USA) using the manufacturer’s instructions.

Isolated RNA was analyzed in a QuantStudio 6 (Thermo Scientific, USA) using TaqMan fast advanced master mix (Thermo Scientific, USA) and appropriate primers/probes (Supplementary Table 1) with the following program: 50 °C for 2 min, 95 °C for 2 min followed by 40 cycles of 95 °C for 3 seconds and 60 °C for 30 seconds. Signals were compared to a standard curve generated using gBlock (Integrated DNA Technologies, IA, USA) corresponding to EEEV FL93-939 E2 protein diluted serially. Viral genome copies per uL were calculated for blood, while viral genome copies in tissue were calculated per gram.

### Hematology and clinical chemistries

Analysis of blood chemistries was performed using a Sysmex XT-2000i analyzer for EDTA collected plasma, or an Olympus AU400 chemistry analyzer for serum.

### Histopathology

Fixed tissues were processed, embedded in paraffin and cut in 5 µm sections. Sections were stained with hematoxylin and eosin (H&E) or left unstained for later analysis via immunofluorescence. Histopathologic lesions identified in tissues were scored semiquantitatively by the same pathologist that performed the necropsies. Lesions were scored based on severity as the lesions being absent (-), minimal (+), mild (++), moderate (+++), or severe (++++).

Immunohistochemical staining (IHC) was performed on 5 um sections of formalin-fixed, paraffin-embedded brain tissue by incubating these sections for 1 h with a primary anti-EEE antibody (Sigma; cat# MAB8754) at a 1:100 concentration. The primary antibody was detected using a MACH3 alkaline phosphatase polymer kit (Biocare Medical, M3M532L) and permanent red. Sections were counterstained with hematoxylin for 5 min. Slides were then imaged with a NanoZoomer S360 slide scanner.

### Statistical analysis

Statistical analyses were performed with GraphPad Prism version 9.5.1(Graphpad). Group comparisons of antibody responses and viral loads were made using a Kruskal-Wallis test with Dunn’s multiple comparisons test. Survival data was analyzed using the Mantel-Cox test. Antibody/viral load relationships were analyzed using the nonparametric Spearman correlation. ELISPOT comparisons were made using the mixed-effects analysis with the Geisser-Greenhouse correction and Tukey’s multiple comparisons test. Hematology and clinical chemistry comparisons were made using a two-way ANOVA.

### Supplementary information

Supplemental Material

## Data Availability

All relevant data are available from the corresponding author upon reasonable request. Source data are provided with this paper.
