# Supplementary material for: MVA-based vaccines are protective against lethal eastern equine encephalitis virus aerosol challenge in cynomolgus macaques
Source: NPJ Vaccines. 2024 Feb 27;9:47. doi: 10.1038/s41541-024-00842-y (PMC10899228; doi:10.1038/s41541-024-00842-y)
Supplement: Supplementary file 1 — Supplemental Material [file 41541_2024_842_MOESM1_ESM.pdf]

Supplementary Table 1: EEEV Primers/Probes

Target	Primer/Probe Designation	Sequence
EEEV E2	EEE 9391	5'- ACA CCG CAC CCT GAT TTT ACA -3'
	EEE 9459c	5'- CTT CCA AGT GAC CTG GTC GTC -3'
	EEE 9414 Probe	5' - /56-FAM/TGC ACC CGG ACC ATC CGA CCT /3BHQ_1/ - 3'

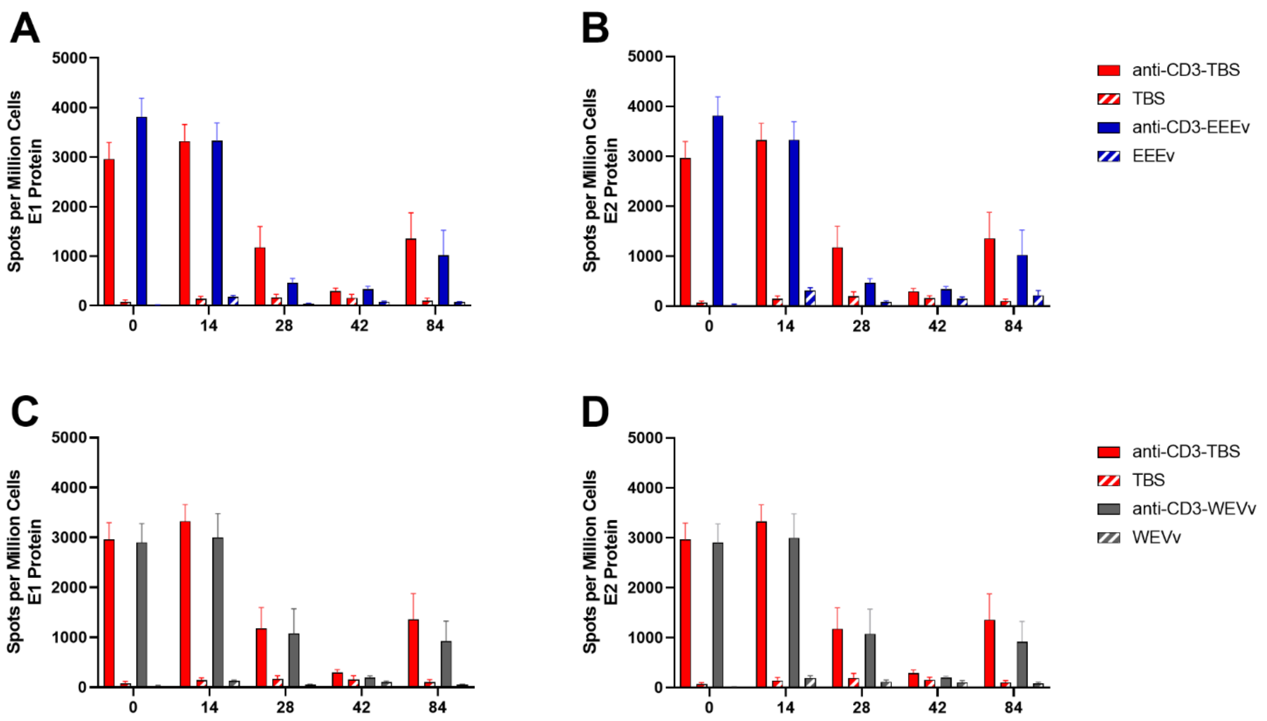

Supplementary Figure 1: Antigen-specific T cell responses post vaccination. Peptide pools in EEEv (A, B) and WEVv (C,D) vaccine formulations representing E1 (A,C) and E2 (B,D) proteins of EEEV were used to assess formation of T-cell responses. Data are shown as mean +/- SE in spots per million cells.

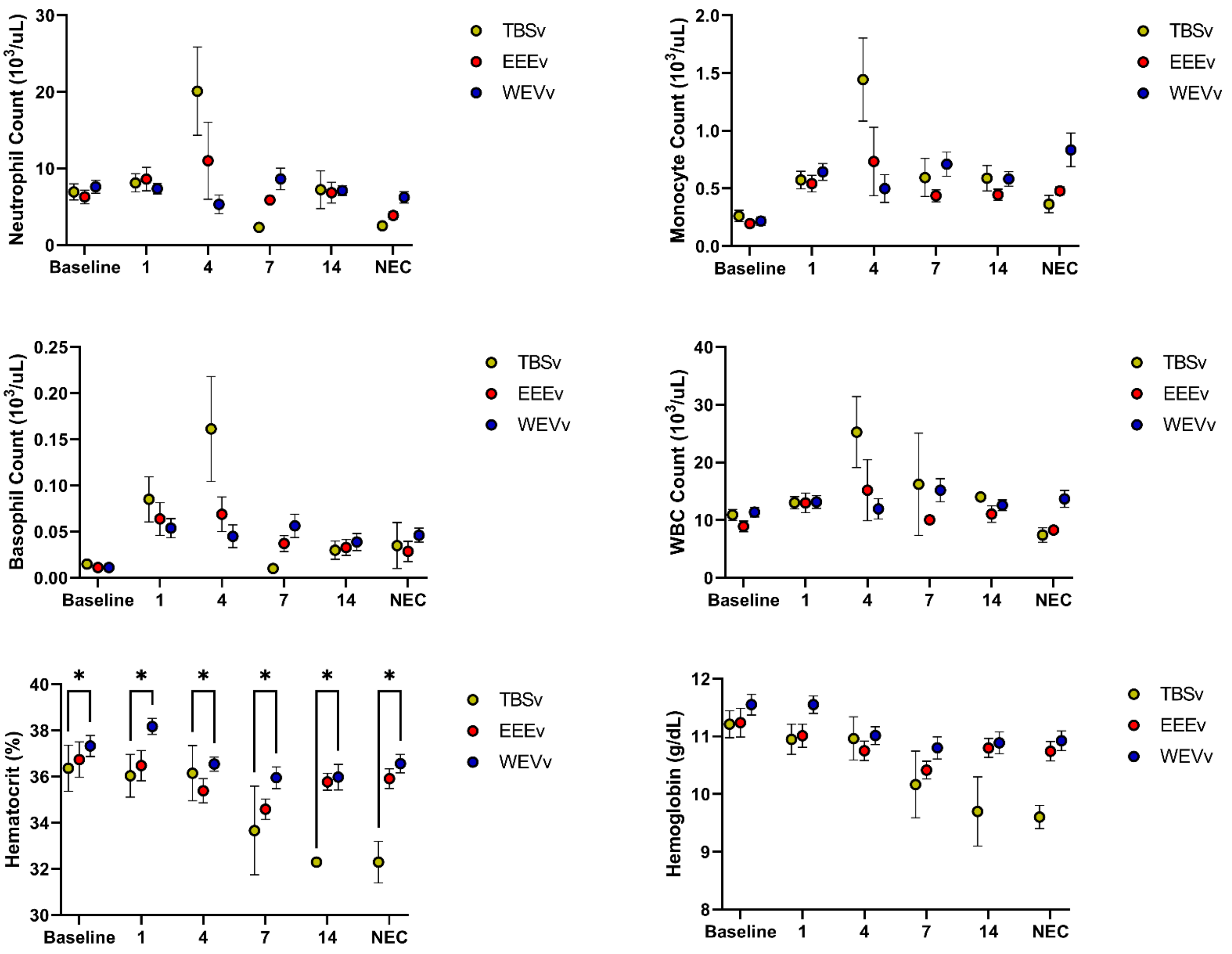

Supplementary Figure 2. Hematology parameters of interest post EEEV challenge. Group comparisons were made using a two-way ANOVA. *: p<0.05. Bars represent SE.

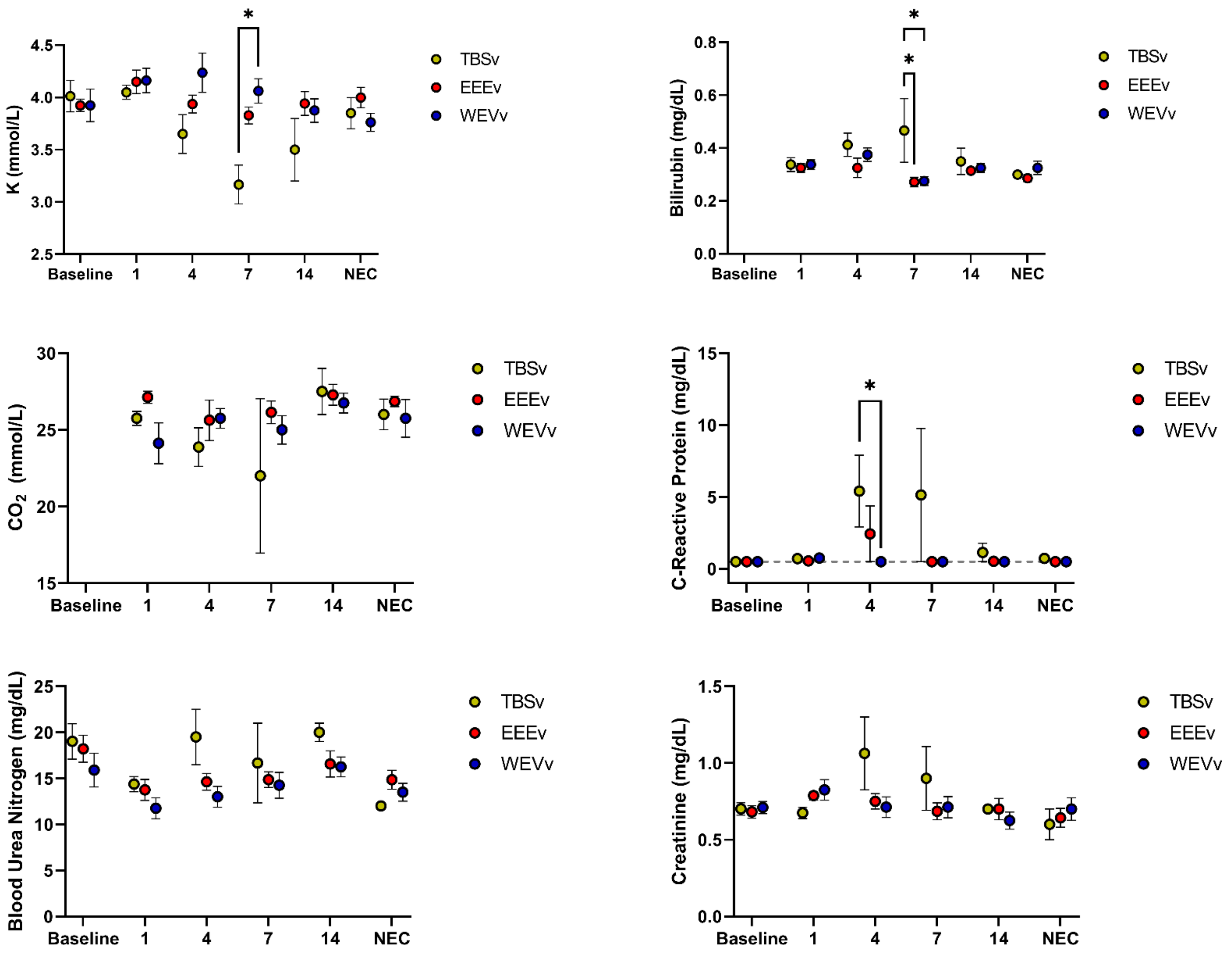

Supplementary Figure 3. Clinical chemistry parameters of interest post EEEV challenge. Group comparisons were made using a two-way ANOVA. *: $p<0.05$. Bars represent standard error.

Supplementary Table 2: Histopathologic findings

	Inflammation												Lymphoid hyperplasia	
Id	Frontal cortex	Parietal cortex	Temporal cortex	Occipital cortex	Brainstem	Cerebellum	Spinal Cord	Left anterior lobe	Left lower lobe	Right anterior lobe	Right middle lobe	Right lower lobe	Bronchial Lymph node	Lung
PB01	-	-	-	-	-	-	-	+	-	+	-	+	+	-
PA97	-	-	-	-	-	-	-	-	+	-	-	+	+	-
PB07	+	-	-	-	-	-	-	+	+	+	+	+	++	-
PA96	-	-	-	-	-	-	-	+	-	+	+	+	-	-
PA93	-	+	+	-	-	-	-	+	+		+	+	+	-
PB05	-	-	-	-	-	+	-	+	+	+	+	-	-	-
PA95	-	-	-	-	-	-	-		+	+	+	-	-	-
PA87	-	-	-	-	-	-	-	+	+	+	-	+	-	-
PA94	-	-	-	-	-	-	-	+	++	-	+	+	-	-
PA90	+	+	+	++	++	+	+	+	+	+	+	+	-	-
PB11	+	+	++	-	-	-	-	+	+	+		-	+	-
PA86	+	-	++	-	-	-	+	+	+	-	+	+		-
PA91	-	-	-	-	+	-	-	+	+	+	+	+	+	+
PA98	-	-	-	-	-	-	-	+	+	+	+	-	+++	-
PB06	-	-	+	-	-	-	-	+	-	-	-	+	+++	+
PB04	-	-	-	-	-	-	-	-	-	++	++	++	++	-
PB08	-	-	-	-	-	-	-	+	-	+	+	-	+	-
PB15	-	-	-	-	-	-	-	-	-	-		+	-	-
PB12	-	+	+	-	-	-	-	++	-	++	++	++	++	-
PB09	-	-	-	-	-	-	-	+	-	-	+	+	-	-
PB02	+	++	++	++	+++	++	++	-	-	++	+	++	-	-
PA92	+++	+++	+++	++	+++	+	++	+	-	-	-	+	-	-
PB10	+	++	++	+	++	-	+++	+	-	+	+	+	-	-
PB14	+++	+++	++	+	++++	++	++	+	-	-	-	-	-	-

-Absent *+Minimal*
++Mild *+++Moderate*

-TBSv
-EEEv
-WEVv
